# Mental health problems among ethnic minorities during public crises: a systematic review of coping strategies during the COVID-19 pandemic

**DOI:** 10.3389/fpubh.2026.1822085

**Published:** 2026-06-01

**Authors:** Naubahar Sharif, Wenjin Chen, Mengyuan Niu

**Affiliations:** 1 Department of Social Science and Policy Studies, The Education University of Hong Kong, Tai Po, Hong Kong, China; 2 Division of Public Policy, The Hong Kong University of Science and Technology, Kowloon, Hong Kong SAR, China

**Keywords:** coping strategies, ecological model, ethnic minorities, mental health, public crises

## Abstract

Public health crises exert a disproportionate psychological burden on ethnic minority populations, exacerbating pre-existing health disparities. Drawing upon the COVID-19 pandemic as a focal case, this systematic review synthesizes the coping strategies employed by ethnic minority communities, organized through the lens of ecological systems theory. Adhering to PRISMA guidelines, 65 peer-reviewed studies published between 2020 and 2023 were analyzed. The synthesis identified a multi-tiered coping response: (1) individual-level cognitive-emotional regulation and faith-based practices; (2) microsystem-level familial and communal support and digital mediation; (3) exosystem-level community governance and health policy; and (4) macrosystem-level cultural values and systemic belief structures. The analysis revealed a pronounced research emphasis on individual and microsystemic adaptations, exposing a critical empirical gap concerning structural and policy-driven interventions. While culturally tailored individual support is essential, these findings underscore the need for future research and policy to prioritize integrated, multilevel frameworks capable of effectively mitigating mental health inequities during future public health emergencies.

## 1 Introduction

Public health emergencies are rarely neutral in their impact; rather, these crises exert a disproportionate psychological burden on ethnic minority populations, acting as catalysts that exacerbate pre-existing systemic inequities (1). While the COVID-19 pandemic catalyzed a global mental health crisis (120–122), it also underscored an urgent need for specialized strategies to mitigate the psychological fallout within ethnic minority communities. Identifying effective coping mechanisms for diverse groups remains a significant challenge, particularly for ethnic minorities who navigate a complex intersection of linguistic barriers, culturally distinct help-seeking behaviors, and pervasive social stigma (2, 3, 112, 118, 119). Such factors often create structural incentives for individuals to conceal psychological distress or underutilize available resources (4). Furthermore, when minority status intersects with low socioeconomic standing, the resulting financial constraints and limited service availability further entrench health disparities, rendering traditional mental health supports largely inaccessible (5, 6).

For the purposes of this review, *ethnic minority* refers to groups that differ from a society’s dominant population in terms of race, ethnicity, or national origin, and who may share distinct cultural practices, languages, or historical experiences (7). Throughout this review, the term *ethnic minority* is used to denote the primary population of interest, with the recognition that their disproportionate mental health burden is partly attributable to structural marginalization.

The COVID-19 pandemic represented a particularly acute manifestation of these vulnerabilities, functioning as a multidimensional stressor characterized by profound uncertainty that impacted individual well-being (8, 108, 111), social cohesion (9, 10), economic security (11, 114), and healthcare infrastructure (12, 104). Given the interconnected nature of these disruptions, it is imperative to investigate mental health promotion through a lens that accounts for individual coping, community-led interventions, and macro-level policy responses (13, 14).

To address these intersecting challenges, the present study conducts a systematic literature review to synthesize mental health promotion strategies utilized by ethnic minority populations during the COVID-19 pandemic, drawing on Bronfenbrenner’s ecological systems theory (15, 16) as the primary analytical framework. The ecological model is particularly suited for this analysis as its multilevel structure mirrors the complex socio-behavioral characteristics of ethnic minority groups (17–19, 115). By facilitating the integration of variables such as socioeconomic status, cultural values, familial dynamics, and broader social contexts, the model captures the reciprocal interactions between the individual and their environment. Previous research has demonstrated the efficacy of this framework in addressing the mental health drivers of vulnerable populations, including youth (105, 116) refugees (20, 117), migrants (21), and sexual minorities (22). Its adaptability makes it an ideal tool for delineating the unique environmental influences that shape the resilience of ethnic minority populations.

Despite growing scholarly attention to ethnic minority mental health, the literature has remained largely preoccupied with barriers to healthcare access (23, 24) or clinical interventions designed for general, non-crisis contexts (25, 26). Consequently, there is a notable empirical gap regarding the active coping strategies employed to improve mental health outcomes for these populations during periods of acute global instability. To address this gap, the review is guided by the following research questions:

1. Which strategies, when analyzed through an ecological lens, proved effective in promoting mental health among ethnic minority individuals during the COVID-19 pandemic?
2. What are the practical and policy-oriented implications of these identified strategies for future public health crisis management?

The subsequent sections delineate the methodology and theoretical underpinnings of the review. By synthesizing key features of the identified strategies, this study provides a comprehensive roadmap for stakeholders to develop culturally sensitive, multilevel frameworks for protecting the mental health of ethnic minorities during future crises.

## 2 Theoretical framework: the ecological systems model

**Figure 1:**
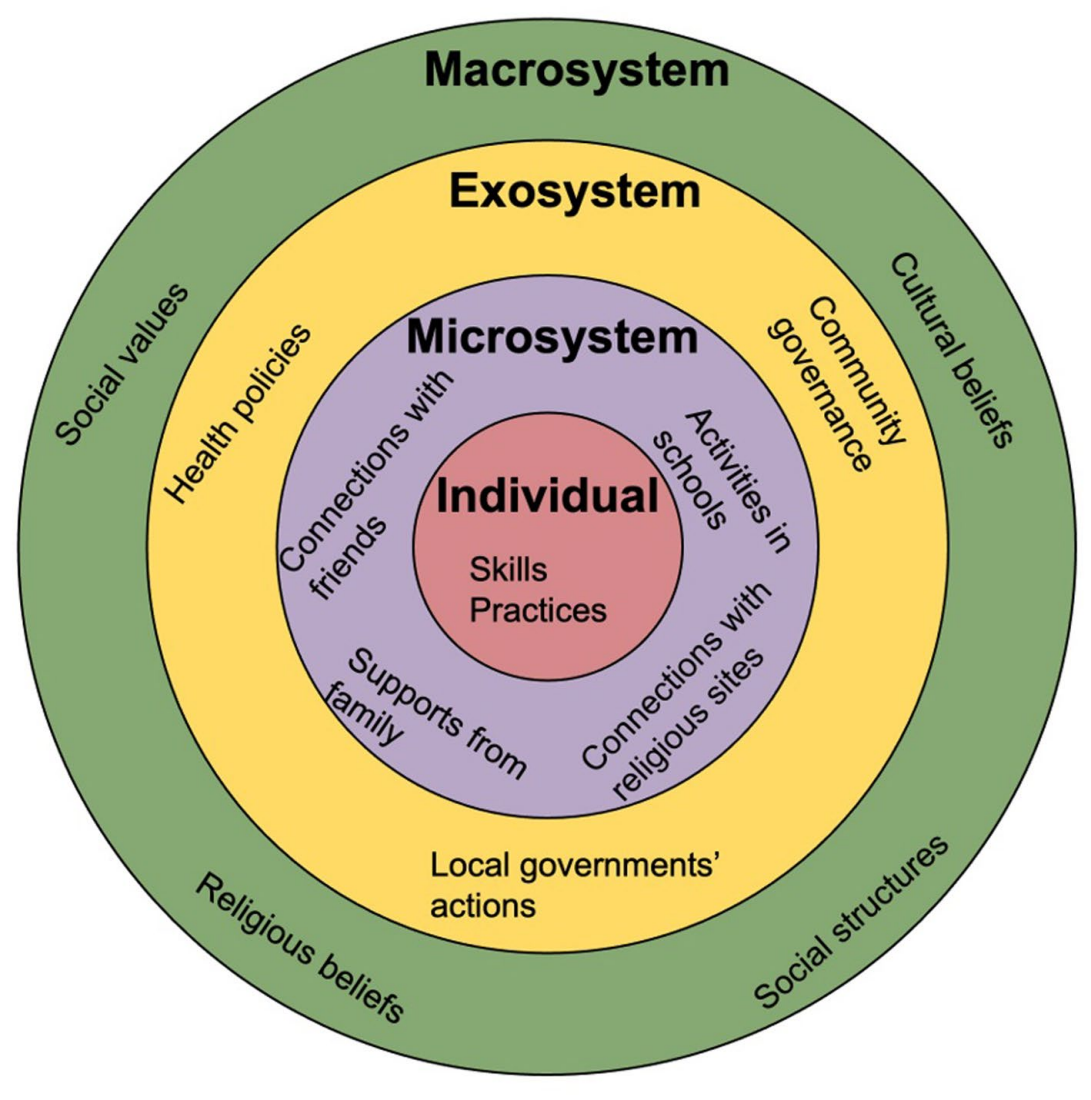
Theoretical conceptualization of the ecological systems model. Modified based on Serdarevic and Chronister (21).

The ecological systems model provides a structured approach to understanding how specific strategies enhance mental health among ethnic minority individuals during the COVID-19 period. This study examines strategies at four levels of the ecological model—individual, microsystem, exosystem, and macrosystem (Figure 1)—each of which operates under distinct assumptions about how environmental contexts shape mental health outcomes.

At the individual level, strategies encompass personal capacities and skills, individually oriented preventive practices, and coping mechanisms (15, 18, 21). At the microsystem level, strategies involve the social activities, interpersonal relations, and social roles that individuals directly engage with in their immediate environment (17). Participating in religious activities at a church or maintaining friendships at school are illustrative examples of microsystem-level phenomena that supported mental health during the pandemic.

At the exosystem level, strategies involve events or activities that span or connect multiple system settings. A defining feature of the exosystem is that ethnic minority individuals are not direct participants in these events, yet are meaningfully affected by them (19). For example, a local government’s decision to expand Medicaid eligibility, or a public health department’s allocation of COVID-19 testing resources to underserved neighborhoods, are exosystem-level events: the individual does not participate in these policy decisions, yet their mental health and access to care are directly shaped by their outcomes (27, 28). Strategies at this level therefore include policies governing the provision of mental health services to ethnic minorities and protective governmental measures designed to safeguard community well-being.

At the macrosystem level, strategies encompass the overarching cultural and ideological features that permeate the individual, microsystem, and exosystem levels. Coping strategies at this level are expressed through social values, cultural beliefs, and broader social structures that collectively shape how ethnic minority communities interpret and respond to adversity (15, 18).

## 3 Method and data

This systematic review follows the Preferred Reporting Items for Systematic Reviews and Meta-Analyses (PRISMA) Statement (29), because the guideline ensures the rigor of a given procedure and enhances the quality of the review process. We follow a PRISMA guideline procedure and specify our search strategy, inclusion criteria, data extraction, and synthesis using the ecological model.

### 3.1 Searches

This systematic review incorporated studies found mainly in four databases: the Web of Science, Scopus, PubMed, and PsycINFO. These databases were chosen because of their diversified bodies of literature in psychology, psychiatry, social science, sociopsychology, and arts and humanities as well as multi-disciplinary literature in broader contexts. The searches were restricted to peer-reviewed articles published in English. To gain a specific understanding of the strategies adopted during the COVID-19 period, the initial search period was set to run from December 2019 to December 2023.^1^

The keyword search was designed to identify strategies employed by ethnic minority populations to manage and mitigate adverse mental health outcomes in the context of the COVID-19 pandemic, using the following search terms:

1. Strategies: “countermeasures” OR “coping strategies” OR “copings” OR “moderators” OR “policy response” OR “policy measures” OR “policy instruments” OR “recovery strategy” OR “protection strategy” OR “promotion strategy.”
2. Ethnic minority population: “Ethnic*” OR “Minority” OR “Race” OR “People of color” OR “Ethnic minorities” OR “Asians” OR “Asians in Europe” OR “Asians in the United States” OR “South Asians in Europe” OR “South Asians in Hong Kong” OR “Koreans in the United States” OR “Chinese in the United States” OR “Bangladeshis in Europe” OR “African Americans in the United States” OR “Blacks” OR “Blacks in Europe” OR “Blacks in the United States.”
3. Mental health: “Mental health” OR “Well-being” OR “Mental Illness” OR “Mental Disorder” OR “Mental problems” OR “Mental Resilience” OR “Depression” OR “Anxiety” OR “Stress” OR “Psycho-.”
4. Research time: “COVID-19” OR “COVID” OR “Coronavirus” OR “SARS-CoV-2” OR “pandemic” OR “epidemic.”

To ensure comprehensive coverage of strategies in our review, this study includes individual-focused coping approaches, policy responses, and common terms for describing strategies, such as “policy instrument,” “countermeasures,” and “recovery strategies.” Meanwhile, the identification of ethnic minorities aims to encompass as many types of ethnic minority groups as possible to identify the common strategies that can be adopted. In addition to such general terms as “ethnic minority,” the search also includes specific ethnic groups commonly found in various countries and regions (30, 31).

### 3.2 Inclusion assessment and data extraction

**Figure 2:**
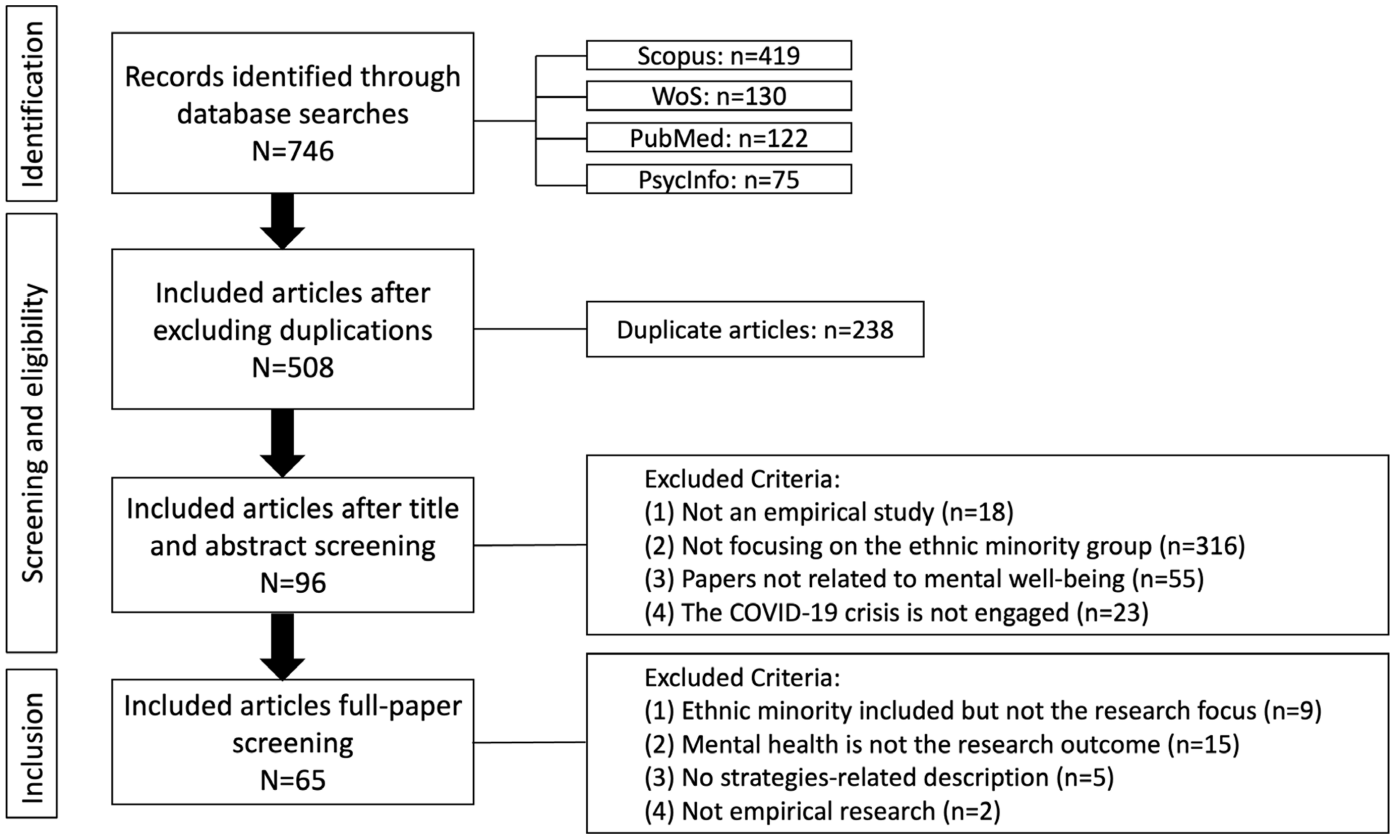
PRISMA flowchart illustrating the selection and identification of included studies.

The initial keyword search identified 746 articles (Figure 2). We managed records through Rayyan, an open-source AI tool that facilitates the systematic review process (32). After removing 238 duplicates, 508 records remained and were screened based on titles and abstracts of articles.

Following our research objectives and questions, the following study characteristics were specified as criteria for this review. First, we restricted the selection to empirical articles published in peer-reviewed journals to ensure the inclusion of high-quality studies that shape and inform scholarly research on strategies for enhancing the mental health of ethnic minority populations. Studies published as book chapters, conference papers, dissertations, and similar documents were excluded (*n* = 18). Second, because our research targeted the ethnic minority population, we excluded articles focused on other minority populations, such as sexual minorities and linguistic minorities (*n* = 316). Third, articles that failed to evaluate mental health outcomes were excluded (*n* = 55). Fourth, articles with time frames that did not include the COVID-19 pandemic were excluded (*n* = 23).

Based on study titles and abstracts, 96 records were included for full-text assessment according to the eligibility criteria (Figure 2). Exclusion criteria during full-paper screening are studies in which ethnic minorities were included only as a reference group or mentioned in a study’s discussion (*n* = 9), mental health was not the research outcome of interest (*n* = 15), or the proposed strategies lack demonstrated effectiveness (*n* = 5). We also excluded systematic review studies and brief reports where findings pertaining to ethnic minority populations’ coping strategies or mental health are rarely included (*n* = 2). Eventually, we identified 65 articles for analysis.

The extracted data, including study setting, sample size, participant characteristics, employed methodologies, identified strategies sorted by ecological system level, and mental health problem outcomes, were processed through Excel. Details on the extracted literature and data can be found in Supplementry Table A1.

## 4 Results

The analyzed articles showcase diverse characteristics across the four system levels of the ecological model as well as research methodologies and ethnic minority identities across multiple countries. Strategies for coping with mental health challenges in all retained papers were coded and categorized into the four levels of the ecological model: individual (*n* = 53, 82%), microsystem (*n* = 34, 50%), exosystem (*n* = 3, 4%), and macrosystem (*n* = 9, 13%).

### 4.1 Individual-level strategies

The majority of the reviewed literature emphasized strategies for enhancing mental health, examined primarily through individual-level analyses. Strategies identified as individual-level strategies utilize behavioral skills, cognitive coping, and religious practices.

#### 4.1.1 Cognitive and emotion-focused coping behaviors

Cognitive coping and emotion-focused coping behaviors were among the most widely documented individual-level strategies adopted by ethnic minority individuals during the COVID-19 period, with consistent evidence reported across diverse populations and cultural contexts. Cognitive coping strategies, which deploy cognitive processes to regulate and manage responses to emotionally stimulating information (33), encompassed a range of specific techniques across the reviewed studies. These included cultivating a sense of meaning in life (34), forming a positive self-concept (35, 36), maintaining positive self-perception (37), identifying alternative courses of action (38), reframing stressors as opportunities for personal growth (39–42), and engaging in cognitive appraisal (43). Despite variation in the specific techniques reported, the studies converged in indicating that these cognitive processes supported the psychological well-being of ethnic minority individuals by fostering more constructive interpretations of stressful situations.

The reviewed studies also comparably highlighted cognitive coping as involving an active problem-identification process. After recognizing pandemic-related academic stress, Asian American students developed cognitive adaptation strategies to manage perceived stress both academically and emotionally (44). Similarly, Black students, whose daily routines were disrupted by COVID-19 restrictions, adopted engagement-oriented strategies such as developing new hobbies to restore structure and purpose to their lives (45). These findings, drawn from different ethnic groups and academic contexts, point to a shared function of cognitive coping: the active reappraisal and reorganization of one’s situation in response to pandemic-induced disruption.

Emotion-focused coping was similarly prevalent and consistent across the reviewed literature, with a broad range of behavioral responses documented across multiple ethnic minority groups. These behaviors included physical activity (46–50), adequate rest (49), maintaining regular daily routines (48, 51), fishing (52), preparing healthy or culturally traditional foods (40, 52, 53), using digital tools and social media to access COVID-19-related information and explore new interests (27, 46, 54, 55), listening to music (53), and engaging in work or other activities as a means of psychological diversion (56). The diversity of these behaviors across ethnic groups and geographic settings suggests that emotion-focused coping was a flexible and culturally adaptable strategy, shaped by individual circumstances and cultural practices while serving a common regulatory function.

Not all reported coping behaviors were adaptive in nature. Chen-Sankey et al. (57) found that smoking was associated with stress reduction and alleviation of boredom during the COVID-19 period, representing a maladaptive coping pattern. This finding stands in contrast to the predominantly positive behaviors documented elsewhere in the literature, underscoring the importance of considering both adaptive and maladaptive coping responses in a comprehensive analysis of ethnic minority mental health during the pandemic.

#### 4.1.2 Building psychological strengths

Building psychological strength was consistently identified across the reviewed studies as a central individual-level strategy for sustaining mental health during the COVID-19 pandemic. The psychological strengths documented spanned multiple constructs, including individual resilience (41, 42, 58–62), a high internal locus of control (63–67), a strong sense of ethnic identity (41, 42, 61, 68–72), self-compassion (73), positive solitude (74), and high self-esteem (75, 76). Despite differences in the specific constructs examined and the populations studied, the studies converged in demonstrating that cultivating internal psychological resources was associated with reduced distress and greater resilience across diverse ethnic minority groups.

Individual resilience was the most extensively documented of these strengths, with consistent findings across multiple ethnic groups and geographic contexts. Defined as a process, capacity, or outcome of successful adaptation in the face of challenging or threatening circumstances (77), resilience was found to serve as a significant protective factor against the psychological impact of the COVID-19 pandemic using standardized measures across studies. Positive associations between resilience and mental health outcomes were reported among Asian Americans, East Asian Americans, Pacific Islanders, Korean Americans, and Palestinians in Israel (41, 42, 58–62), indicating that the protective function of resilience was not specific to any single cultural or national context.

A high internal locus of control was similarly associated with better mental health outcomes, with comparable findings reported across culturally distinct groups. Filipino Americans who perceived their behaviors as driven by personal decisions and effort reported greater confidence in their capacity to manage pandemic-related adversity, which was associated with reduced stress and anxiety (67). A survey study of an Arab minority group in Israel likewise revealed a negative correlation between internal locus of control and feelings of threat and loneliness during the COVID-19 period (63). The consistency of this finding across Filipino American and Arab minority samples, despite their cultural differences, suggests that a sense of personal agency may function as a broadly applicable psychological resource during public health crises.

Embracing one’s ethnic identity also emerged as a consistent protective factor across the reviewed literature. Cheah et al. (69) found that Chinese Americans with a high level of bicultural identity reported fewer mental health difficulties attributable to racial discrimination during the pandemic. Similarly, Bilewicz et al. (68), studying ethnic minorities in Poland, found that a strong sense of ingroup interconnectedness was associated with reduced stress and anxiety. These findings, derived from distinct ethnic minority populations in different national contexts, point to a shared mechanism: a positive relationship with one’s ethnic identity may buffer against the psychological consequences of discrimination and social marginalization during periods of crisis.

Beyond resilience, locus of control, and ethnic identity, the reviewed studies also highlighted the protective value of several additional psychological strengths, though with more limited evidence bases. Self-compassion was associated with reduced mental health burden among ethnic minority groups during the pandemic (73). Positive solitude was linked to lower levels of anxiety, depression, and trauma among Black and mixed-ethnicity individuals (74). Higher self-esteem was associated with fewer depressive symptoms among Asian Americans (78) and with reduced psychological distress and a lower somatic symptom burden among African Americans with histories of depression and anxiety (75). Although each of these findings derives from a single study, their consistent alignment, all demonstrating a positive association between internal psychological resources and mental health, strengthens the broader pattern evident across this subsection.

#### 4.1.3 Religious practices

Religious practices at the individual level were among the most consistently documented coping strategies across the reviewed studies, with comparable findings reported across Christian and Muslim communities in multiple countries. Ethnic minority individuals drew upon a wide variety of faith traditions and religious behaviors to manage the psychological demands of the COVID-19 pandemic, and the protective function of these practices appeared to operate similarly across culturally distinct groups.

Among Christian communities, faith-based practices were repeatedly identified as a primary source of emotional regulation and psychological support. Australian Africans of Christian faith emphasized the centrality of religion- and faith-based well-being practices in managing emotional changes during the pandemic, describing prayer, fasting, listening to gospel music, and following religious leaders on social media as forms of spiritual self-care that provided relief during periods of low mood and depression (47). Similarly, individuals from Black, Asian, and Minority Ethnic (BAME) groups reported that reading the Bible and drawing strength from their belief in God helped them endure pandemic-related mental stress (79). Among Black Americans more broadly, personal religious and spiritual practices, including prayer, meditation, and virtual connection to church communities, were widely endorsed as coping resources (53), a pattern also reported among Black students and African American women, who used prayer and biblical scripture to navigate the psychological demands of the COVID-19 period (80, 81). “Across these studies, the specific practices varied, yet their shared function of providing comfort, structure, and a sense of spiritual connection during adversity remained consistent.

Comparable findings were reported among Muslim ethnic minority communities, suggesting that the protective role of individual religious practice extended across faith traditions. Pakistani Muslims in the United Kingdom reported increasing the time devoted to prayer and Quranic reading during the pandemic, describing these practices as a source of strength and inner peace in the face of uncertainty (82). Muslim ethnic minorities in Northwest England similarly reported that prayer and supplication were effective in reducing psychological distress during the COVID-19 period (83). The convergence of these findings across Christian and Muslim communities, and across multiple national contexts, indicates that individual religious practice served as a broadly effective and culturally embedded strategy for managing pandemic-related psychological distress among ethnic minority populations.

### 4.2 Microsystem-level strategies

Studies conducted at the microsystem level predominantly examined how ethnic minority individuals connected with others and community groups to alleviate mental health challenges during the pandemic. The key findings indicated that receiving social support from family and friends, actively engaging with community organizations for assistance, and maintaining or developing social relationships through the Internet can effectively mitigate mental health challenges during the pandemic.

#### 4.2.1 Social support from family and friends

Across the reviewed studies, social connectedness through family and friendship support emerged as the most consistently identified microsystem-level strategy for mitigating adverse mental health effects during the COVID-19 period. This convergent pattern was documented across a wide range of ethnic minority groups and geographic contexts, including Latinx Americans, East Asian Americans, African American women, international students in Poland, Chinese international students, older Chinese immigrants in the United States, and Asians in Australia (38, 62, 84–89). The breadth and cross-cultural consistency of this evidence suggests that the protective role of family and social support operated as a universal resource that transcended cultural and national boundaries. Across these studies, support encompassed practical assistance, emotional encouragement, and informational guidance, and a positive relationship between these forms of support and mental health outcomes was consistently reported regardless of the ethnic group or setting studied.

The reviewed articles were also comparable in documenting the specific mechanisms through which family support operated, with studies across different populations identifying similar pathways. Hassoun Ayoub et al. (90) and Huang and Tsai (91) reported that Asian American parents and Black American mothers increased quality time with their children and families during the pandemic, which was associated with significant reductions in COVID-19-related stress. Similarly, African Americans drew on family assistance to reestablish daily routines and a sense of normalcy, a strategy that helped buffer pandemic-induced stress (40). Among Filipino Americans managing type-2 diabetes, whose self-management demands were intensified by pandemic conditions, family-sourced emotional support and encouragement proved instrumental in mitigating resulting negative emotions (67). Virtual family contact served a comparable protective function in a different cultural context: transitional career employees with Pakistani, Syrian, African, and South American backgrounds living in Canada reported that mutual encouragement exchanged through digital platforms helped them navigate work-related stressors (51). Notably, while the specific form of support varied across groups—ranging from increased in-person family time to virtual contact—the direction and nature of the relationship between family support and mental health was consistent across all studies. Taken together, these findings indicate that family support, whether delivered in person or virtually and whether practical, emotional, or informational in nature, served as a robust and broadly applicable buffer against pandemic-related psychological distress across diverse ethnic minority communities.

#### 4.2.2 Community involvement

In addition to family and friendship networks, engagement with broader community structures was consistently found to have a positive impact on mental health during the COVID-19 pandemic. The communities involved were diverse, encompassing residential communities (36, 37, 92, 93), religious organizations (81), and interest-based groups (46, 94). Across these varied community types, a common pattern emerged: community engagement provided ethnic minority individuals with practical assistance, reliable health information, and emotional support, all of which contributed to reducing psychological distress during the pandemic.

This convergence was evident across culturally distinct groups. In Hispanic residential communities, social bonds were strengthened during the pandemic, with community members offering each other practical help such as food sharing, which was associated with reduced anxiety and depression (36). Among African Americans in Houston, community institutions, including churches, universities, employers, public health departments, and residential agencies, functioned as trusted sources of reliable health information when government messaging was perceived as inconsistent, thereby helping to alleviate concern and frustration (27). These findings suggest that the value of community engagement was not limited to emotional support but extended to addressing informational and material needs during the crisis.

The reviewed studies also indicated that ethnic minorities were more likely to engage with communities to which they felt a sense of belonging or shared identity. Black youth in the United States reported feeling better understood within religious communities than in school settings, and this sense of belonging facilitated more constructive management of negative emotions (81). Similarly, Asian American students who formed voluntary peer groups based on shared ethnic identity showed greater willingness to access mental health services, as the shared identity lowered barriers to help-seeking and reduced pandemic-related psychological distress (94). Together, these findings indicate that cultural congruence and shared identity were important facilitating factors in community-based mental health support.

#### 4.2.3 Facilitating social connectedness through internet engagement

Across the reviewed studies, Internet use emerged as a consistent strategy for maintaining social connections and mitigating the psychological consequences of pandemic-related social restrictions among ethnic minority individuals, with evidence spanning students, women, essential workers, and older adults. Despite variation in the specific platforms and modalities used, all relevant studies reported beneficial effects on social connectedness and emotional well-being.

Black college students, for instance, reported conducting virtual check-ins with peers to provide mutual comfort and support when in-person contact was unavailable (45). Among essential workers experiencing stress related to caregiving and occupational responsibilities, videoconferencing platforms enabled social networking and communication with new contacts, which helped ease frustration (95). Among older African American adults, who faced particular risks of isolation during the COVID-19 lockdown, learning to use mobile phones and applications such as Zoom and Facebook Live to host virtual meetings and social gatherings proved effective in sustaining social connections and reducing feelings of loneliness (27). The consistency of these findings across demographically diverse groups suggests that digital tools served as a broadly accessible and effective means of preserving social cohesion among ethnic minority populations during the pandemic.

### 4.3 Exosystem-level strategies

Although the exosystem level was the least represented in the reviewed literature, the studies identified at this level consistently highlighted the critical role of health policy, community governance, and government leadership in shaping mental health outcomes among ethnic minority populations during the COVID-19 period. These findings underscored the importance of structural influences on mental health that extend beyond individual and interpersonal factors.

#### 4.3.1 Health policy

The health policy evidence in the reviewed articles focused primarily on Medicaid, a public health insurance program in the United States that provides essential healthcare services to low-income individuals and families. Oyeka and Wehby (28) examined the impact of Medicaid expansion on mental health outcomes among non-Hispanic Black and other non-Hispanic non-White populations, finding that expanded eligibility was associated with improved mental health among low-income adults. This finding points to the potential of structural policy interventions to reduce mental health disparities among ethnic minority populations during public health crises.

#### 4.3.2 Community governance

The included articles addressing community governance examined the capacity and leadership of local government officials in supporting the mental health of ethnic minority populations during the COVID-19 pandemic. Specifically, the studies assessed the governance role of police officers at the neighborhood level and the leadership of a city mayor and a county judge.

Ngo et al. (96) measured community perceptions of police responsiveness and crime management during the pandemic, finding a positive association between favorable perceptions of local policing and improved mental health outcomes among community members. This finding suggests that the perceived quality of local governance extended beyond public safety to influence the psychological well-being of ethnic minority residents.

Adepoju et al. (27), drawing on group interviews with African Americans, found that culturally responsive leadership at the local government level was associated with reduced mental health burden. Participants expressed appreciation for two specific actions taken by a mayor and a county judge: the cancelation of a widely attended annual rodeo event to limit viral transmission, and the proactive translation of all COVID-19-related public health information into both English and Spanish. These measures were perceived by ethnic minority citizens as evidence that their health and cultural needs were recognized by political authorities, which fostered a sense of inclusion and contributed to alleviating pandemic-related psychological distress. Together, these findings indicate that responsive and culturally sensitive local governance can serve as a meaningful structural resource for ethnic minority mental health during public health emergencies.

### 4.4 Macrosystem-level strategies

At the macrosystem level, religious beliefs and social values were identified as critical cultural and ideological forces that supported mental health during the COVID-19 pandemic. Across the included studies, these macrosystem-level influences operated not as individual coping behaviors but as shared cultural frameworks that shaped collective resilience and communal responses to adversity.

#### 4.4.1 Religious beliefs

Unlike the individual-level religious practices discussed in Section 4.1, religious beliefs at the macrosystem level functioned as a foundational cultural and ideological force shaping collective trust and communal coping during the pandemic. This pattern was evident across multiple faith traditions. Within Muslim communities, belief in Allah and the concept of predestination provided a sense of comfort and psychological protection (48, 82), and trust in divine sovereignty was associated with greater peace of mind in the face of uncertainty (52). Among Christian communities, a shared belief in divine protection facilitated collective trust within families and religious networks, serving as a buffer against anxiety during the pandemic (95). Parker et al. (97) further found that a positive image of God was associated with greater strength, hope, confidence, calmness, contentment, and motivation among Black adolescents in the United States, supporting psychological resilience throughout the COVID-19 period. The consistency of these findings across Muslim and Christian communities suggests that macrosystem-level religious beliefs provided a broadly applicable framework for collective meaning-making during the crisis.

#### 4.4.2 Social values

While the reviewed studies predominantly identified religious beliefs as a macrosystem-level resource for mental health, one included article drew attention to the distinct protective role of culturally specific social values. Tolentino et al. (67) found that Filipino respondents drew upon a deeply rooted sociocultural value encapsulated in the phrase “whatever will be, will be,” an expression of acceptance and fatalistic resilience, to sustain determination and equanimity in the face of pandemic-related uncertainty. Although this finding is limited to a single study and one ethnic group, it suggests that culturally embedded worldviews and value systems may function as meaningful macrosystem-level resources for psychological resilience, and that their role in crisis coping warrants broader investigation across other ethnic minority communities.

## 5 Discussion

This systematic review identified a diverse array of strategies utilized by ethnic minority populations to mitigate the psychological impact of the COVID-19 pandemic. The analysis through the lens of Bronfenbrenner’s ecological model revealed that while coping mechanisms were prevalent at the individual and microsystem levels, there remained a critical dearth of evidence regarding the efficacy of exosystemic social policies and structural interventions.

### 5.1 Individual and microsystemic resilience: the role of faith and connectivity

At the individual level, religious practices, cognitive-emotional regulation, and psychological resilience emerged as primary buffers against mental distress. The prominence of religious practice as a culturally significant coping mechanism aligns with existing gerontological and oncology literature (98, 99), suggesting that for many ethnic minorities, faith functions as a fundamental cognitive framework for crisis management (98, 109).

At the microsystem level, the findings challenge traditional assumptions regarding ethnic minorities’ reluctance to engage with communal resources due to stigma (2). Instead, the findings indicated that residential, social, and religious communities provided vital emotional and practical scaffolding. Furthermore, digital mediation (Internet use) played a transformative role in maintaining social cohesion. While previous research often highlights the “digital divide” or negative psychological outcomes of screen time (100), the findings suggested that for marginalized groups, digital platforms served as essential, scalable tools for circumventing physical isolation and accessing cost-effective support (101, 110, 113).

### 5.2 The exosystemic gap: a call for policy integration

A significant finding of this review is the empirical “silence” at the exosystem level. Very few studies, largely concentrated in the United States, have examined the responsiveness of local governance or assessed the effects of specific health policies on the mental health of minority populations. This gap suggests that current crisis management often defaults to individualizing resilience rather than implementing systemic protections. To move forward, policies must transcend traditional clinical frameworks by fostering formal collaborations between governmental health bodies and community-religious organizations (94). Such partnerships are essential for reducing access barriers and ensuring that outreach is culturally congruent (81).

### 5.3 Macrosystemic influences: religion as a cultural blueprint

The macrosystem-level analysis underscores that religious belief is not merely an individual behavior but a foundational cultural “blueprint” that shapes collective mindsets and social interaction patterns (102). In many ethnic minority communities, the macrosystemic influence of religion provides a shared language for suffering and hope, which in turn drives behaviors at the micro and individual levels. Recognizing religion as a structural component of social culture is, therefore, vital for designing inclusive mental health frameworks.

Based on this synthesis, two primary implications emerge for future crisis intervention and academic inquiry. First, there is an urgent need for rigorous, policy-driven research at the exosystemic level to evaluate the scalability of digital mental health platforms and the efficacy of formal community-religious partnerships (107). Future studies must assess how structural interventions can be designed to support and amplify, rather than replace, existing grassroots resilience and informal support networks. Second, while the significance of culture is widely acknowledged in the literature (103, 106), it remains insufficiently integrated into systematic theoretical frameworks; therefore, future research should prioritize the development of “culturally grounded” ecological models. Such frameworks would ensure that interventions are not merely generic but are specifically tailored to the unique religious, social, and historical contexts of ethnic minority populations. By bridging the gap between individual coping and systemic policy, stakeholders can move toward a more proactive and equitable approach to mental health protection during public health emergencies.

## 6 Conclusion

The COVID-19 pandemic has served as a critical stress test for public mental health systems, exposing the unique vulnerabilities and strengths of ethnic minority communities. By utilizing an ecological framework, this review has mapped a hierarchy of coping strategies—ranging from individual faith and digital connectivity to communal support. However, the prevailing research bias toward individual-centric strategies highlights a missed opportunity for systemic reform.

The present review advocates for a paradigm shift toward multilevel, culturally tailored frameworks that integrate individual resilience with robust policy-level support. Addressing the specific cultural and religious needs of ethnic minorities is not merely a matter of inclusivity; it is a prerequisite for effective crisis management. Ultimately, bridging the gap between individual coping and systemic policy will ensure a more equitable and resilient mental health landscape for vulnerable populations in future public health emergencies.

## Data Availability

The original contributions presented in the study are included in the article/Supplementary material, further inquiries can be directed to the corresponding author.

## Footnotes

1 In May 2023, the WHO declared an end to the COVID-19-related global public health emergency. Given the typical delay in the publication of scholarly articles, the search period was extended to December 2023.
