# Supplementary material for: Mental health problems among ethnic minorities during public crises: a systematic review of coping strategies during the COVID-19 pandemic
Source: Front Public Health. 2026 Jun 1;14:1822085. doi: 10.3389/fpubh.2026.1822085 (PMC13265332; doi:10.3389/fpubh.2026.1822085)
Supplement: Supplementary file 1 [file Data_Sheet_1.PDF]

Appendix

Table A1: Characteristics of included studies

No.	Title	Authors	Year	Methodology	Study setting	Participants (Number & ethnicity & Other particular identity)	Protective strategies	Impact on mental well-being
1	Resilience and Risk Factors Predict Family Stress Among Married Palestinians in Israel During the COVID-19 Pandemic	Hassan-Abbas, NM	2022	Online survey	Israel	N=480<br>Ethnicity: Palestinians in Israel	**Individual:** Individual resilience<br>**Micro:** Family resilience;	Marital stress, parental stress, and financial stress
2	Demographic and COVID-19-Related Factors Associated with Depressive and Anxiety Symptoms Among African American and Latina Women in a Midwestern State	Lara-Cinisomo, S and Melesse, B and Mendy, ME	2023	Online survey	USA	N=1037<br>Ethnicity: Latina and African American (AA)<br>Other particular identity: Women	**Individual:** Praying to cope	Anxiety and depressive
3	Purpose in the Pandemic: Fear of COVID-19, Hopelessness, Meaning in Life, and Suicidal Thoughts Among Two Samples of Black Americans	Goodwill, J.R.	2023	Online survey	USA	Sample 1 N= 489 & Sample 2 N= 794<br>Ethnicity: Black American	**Individual:** Presence of meaning in life	Suicidal ideation
4	Psychological Well-Being in Asian and Asian American University Students: Impacts of Discrimination During the COVID-19 Pandemic	Rivera Juarez, A.G. and Prichard, J.R. and Berg, S.S.	2023	Quantitative survey	USA	Sample 1 N=4,759 & Sample 2 N=1,692<br>Ethnicity: East Asian, Southeast Asian, South Asian, and other Asian	**Individual:** Well-rested, exercising	Psychological distress

5	A qualitative study on lived experience of self-harm in South Asians in the UK: From reasons to recovery	Özen-Dursun, B. and Panagioti, M. and Alharbi, R. and Giles, S. and Husain, N.	2023	Semi-structured interview	UK	N=11<br>Ethnicity: South Asian	**Individual:** Having a routine, go diet, regular exercise, and sports;<br>**Micro:** Seeking for professional help; improve social relationships<br>**Macro:** Faith and spirituality, linking to God, Allah	Depression, anxiety, sadness, guilt, anger, feel disheartened, confusion
6	Haitian and Haitian American experiences of racism and socioethnic discrimination in Miami-Dade county: At-risk and court-involved youth	Cela, T. and Marcelin, L.H. and Waldman, R. and Dembo, R. and Demezier, D. and Clement, R. and Arcayos, A. and Santisteban, D. and Jean-Gilles, M. and Hogue, A.	2023	Observation and interview	USA	N=35<br>Ethnicity: Black and Haitian	**Individual:** Positive self-concept	Fear, hopelessness, and chronic stress reactions
7	Experience of Ethnic Discrimination, Anxiety, Perceived Risk of COVID-19, and Social Support among Polish and International Students during the Pandemic	Bokszczanin, A. and Gladysh, O. and Bronowicka, A. and Palace, M.	2023	Online survey	Poland	N=105<br>Ethnicity: Ukrainian, Vietnamese, German, Belarusian, Ethiopia, Nigeria, Greece, Mexico, Iran, Palestine, Czechia, and Russia<br>Other particular identity: International students	**Micro:** Perceived social support from family, friends, and significant others	Anxiety
8	Effects of the Affordable Care Act Medicaid Expansions on Mental Health During the COVID-19 Pandemic in 2020-2021	Oyeka, O. and Wehby, G.L.	2023	Longitudinal survey	USA	N=128074<br>Ethnicity: Black non-Hispanic, other non-Hispanic, Hispanic	**Exo:** Medicaid expansion for non-Hispanic Black and other non-Hispanic non-White individuals	Stress, depression, and emotional problems
9	Psychological distress and anxiety in Arab refugees and migrants during the COVID-19 pandemic in Germany	Jumaa, J.A. and Bendau, A. and Ströhle, A. and Heinz, A. and Betzler, F. and Petzold, M.B.	2023	Mix-method: Online survey and qualitative interview	German	Online survey: N=85<br>Qualitative interview: N=10<br>Other particular identity: Arabic-speaking refugees in Germany	**Individual:** Go fishing and doing Muslim dishes<br>**Macro:** Link to God	Anxiety

10	Anti-Asian racism related stigma, racial discrimination, and protective factors against stigma: a repeated cross-sectional survey among university students during the COVID-19 pandemic	Boden-Albala, B. and Ding, X. and Ryan, N. and Goodman, S. and Wing, J. and Runnerstrom, M.G. and Gutierrez, D. and Gibbs, B. and Robb, J.M. and Drum, E.	2023	Longitudinal survey	USA	N=1,359 & N=1196<br>Ethnicity: Asian America and Pacific Islanders, Hispanic, African American	**Individual:** Maintaining a healthy lifestyle, thinking positively about oneself<br>**Micro:** Living with neighbors/roommates	Stigma
11	Social Support and Technology Use and Their Association with Mental and Physical Health During the COVID-19 Pandemic Among Asian Americans: The COMPASS Cross-sectional Study	Park, L.G. and Meyer, O.L. and Dougan, M.M. and Golden, B. and Ta, K. and Nam, B. and Tsoh, J.Y. and Tzuang, M. and Ta Park, V.M.	2023	Quantitative survey	USA	N=4631<br>Ethnicity: Asian American and Pacific Islanders	**Micro:** Social support from relatives/friends	Depressive and anxiety
12	Psychological distress and everyday discrimination among Chinese international students one year into COVID-19: a preregistered comparative study	Bi, K. and Yeoh, D. and Jiang, Q. and Wienk, M.N.A. and Chen, S.	2023	Cross-sectional online survey	USA	N=381<br>Ethnicity: Chinese<br>Other particular identity: Chinese international students	**Micro:** High perceived social support	Depression and anxiety
13	Paradoxical Effects of Ethnic Identification on Threat and Anxiety During COVID-19 Pandemic. A Study of Ethnic Minority and Immigrant Groups	Bilewicz, M. and Mirucka, M. and Olko, J.	2022	Online survey	Poland	N=2674<br>Ethnicity: Kashubian, Lemko, Silesian, Ukrainian in Poland	**Individual:** Individual feelings of attachment and similarity to other ethnic members	Anxiety, COVID-19 threat
14	The Psychological Well-Being of Older Chinese Immigrants in Canada amidst COVID-19: The Role of Loneliness, Social Support, and Acculturation	Su, C. and Yang, L. and Dong, L. and Zhang, W.	2022	Online survey	Canada	N=168<br>Ethnicity: Chinese<br>Other particular identity: older Chinese immigrants	**Micro:** Perceived social support from family, friends, and significant others	Psychological well-being

15	Asian American Parents' Experiences of Stress, Discrimination, and Mental Health During COVID-19	Huang, C.Y. and Tsai, W.	2022	Quantitative survey	USA	N=166<br>Ethnicity: Asian American<br>Other particular identity: parents of children ages 2-19 years	**Micro:**<br>Increased quality time with their family	COVID-19-related stress
16	COVID-19-Related Racial Discrimination on Asian Australians: An Evaluation of Symptoms of Psychological Distress, Social Support, and Acculturation	Lim, S.Y. and MacDonald, J.B.	2022	Online survey	Australia	N=113<br>Ethnicity: Southeast Asian, East Asian, South Asian	**Micro:** Social support from significant others and family	Anxiety and stress symptoms
17	Do social isolation and neighborhood walkability influence relationships between COVID-19 experiences and wellbeing in predominantly Black urban areas?	Finucane, M.L. and Beckman, R. and Ghosh-Dastidar, M. and Dubowitz, T. and Collins, R.L. and Troxel, W.	2022	Longitudinal survey	USA	2020 N=837 & 2018 N=327<br>Ethnicity: Black	**Micro:** Neighbourhood walkability and social connection	Psychological distress
18	The impact of COVID-19 social distancing and isolation recommendations for Muslim communities in North West England	Hassan, S.M. and Ring, A. and Tahir, N. and Gabbay, M.	2021	Qualitative interview	UK	N=22<br>Ethnicity: Muslim ethnic minorities	**Individual:** coping through prayers and supplication<br>**Macro:** Belief in fate and death as Allah's (God) decree; belief that COVID-19 is a test	Psychological distress
19	Self-Perception of changes in lifestyle and wellbeing associated with social distancing during COVID-19 pandemic among medical students (the study in Lviv, Ukraine)	Muzyka, I. and Belka, B. and Ostrovska, Y. and Zayachkivska, O.	2021	Online survey	Ukraine	N= 273<br>Ethnicity: Asian, Caucasian, African, Hispanic, and multiracial ethnic	**Individual:** doing exercise regularly	Stress

20	Exploring changes in cigar smoking patterns and motivations to quit cigars among black young adults in the time of COVID-19	Chen-Sankey, J.C. and Broun, A. and Duarte, D.A. and Ajith, A. and Jewett, B. and Smiley, S.L. and Mead-Morse, E.L. and Guy, M.C. and Choi, K.	2020	In-depth phone interview	USA	N=40<br>Ethnicity: Black<br>Other particular identity: Black young adults ageing 21 to 29	**Negative coping - Individual:** smoked more cigarillos and blunts	Stress and boredom
21	Mental Health Concerns During COVID-19: An Observational Study Among a Predominantly Black Community in New York City	Ngo VK, Vu TT, Punter MA, Levine D, Mateu-Gelabert P, Borrell LN.	2023	Online survey	USA	N=393<br>Ethnicity: Black	**Exo:** Better community perception of the police	PTSD, depression, and anxiety
22	Chinese American adolescents' experiences of COVID-19 racial discrimination: Risk and protective factors for internalizing difficulties	Cheah, Charissa S. L. and Zong, Xiaoli and Cho, Hyun Su and Ren, Huiguang and Wang, Suqing and Xue, Xiaofang and Wang, Cixin	2021	Online survey	USA	N=211<br>Ethnicity: Chinese American<br>Other particular identity: adolescents and parents	**Individual:** High level of bicultural identity integration harmony and blendedness;	Internalizing difficulties
23	Effects of COVID-19, discrimination, and social support on Latinx adult mental health	Held, Mary Lehman and First, Jennifer M. and Huslage, Melody	2022	Online survey	USA	N=264<br>Ethnicity: Latinx	**Micro:** Social support	Depression, posttraumatic stress
24	Experience of and worry about discrimination, social media use, and depression among Asians in the United States during the COVID-19 pandemic: Cross-sectional survey study	Pan, Shuya and Yang, Chia-chen and Tsai, Jiun-Yi and Dong, Chenyu	2021	Web-based, cross-sectional survey	USA	N=209<br>Ethnicity：Asian	**Individual:** Less worry about discrimination	Depression

25	Psychosocial Stressors and Coping Strategies Among African Americans During Early Stages of the COVID-19 Pandemic: a Qualitative Study	Gillyard, T. and Davis, J. and Parham, I. and Moss, J. and Barre, I. and Alexander, L. and Cunningham-Erves, J.	2023	Qualitative, semi-structured interviews	USA	N=62<br>Ethnicity: African American<br>Other particular identities:6 parents, 15 young adults, 16 essential workers, and 15 individuals with underly ing medical conditions	**Individual:** meaning making, reframing their stress as opportunities for growth and development; strength spiritual connection; cook healthier meals or be more mindful about their diet; cognitive coping strategies (e.g., positive thinking, limited interaction with media or social media platforms, and even formal therapy);<br><br>**Micro:** getting assistance from family;	stress, anxiety, and/or depression, fear of getting infected,
26	Asian American University Students' Adjustment, Coping, and Stress during the COVID-19 Pandemic	Hwang, J. and Ding, Y. and Chen, E. and Wang, C. and Wu, Y.	2023	Quantitative survey	USA	N=103<br>Ethnicity: Asian American<br>Other particular identity: university students	**Individual:** Capability of academic adjustment, personal emotional adjustment	perceived stress
27	Experiences of Filipino Americans with Type 2 Diabetes during COVID-19: A Qualitative Study	Tolentino, D.A. and Roca, R.P.E. and Yang, J. and Itchon, J. and Byrnes, M.E.	2023	Semi-structured interview	USA	N=19<br>Ethnicity: Filipino	**Individual:** Having food; adapting to a changing environment by adjusting their lifestyle; emotion-focused coping; growth and locus of control<br>**Micro:** Utilizing social and familial support and resources; technology use for increasing social connectedness<br>**Macro:** Sociocultural value-"whatever will be, will be."	Stress, feelings of depression, loneliness, isolation, or anxiety

28	"I Have a Wish": Anti-Asian Racism and Facing Challenges Amid the COVID-19 Pandemic Among Asian International Graduate Students	Dong, F. and Hwang, Y. and Hodgson, N.A.	2023	Interview	USA	N=22<br>Ethnicity: Asian<br>Other particular identity: Asian International graduate students	**Micro:** Participate in student-run programs	Stress
29	Impact of the COVID-19 pandemic on young people from black and mixed ethnic groups' mental health in West London: A qualitative study	Lenoir, R. and Wong, K.K.-Y.	2023	Interview	USA	N=10<br>Ethnicity: Black and mixed ethnic group	**Individual:** Positive solitude<br>**Micro:** see friends when social restrictions lifted	Anxiety, depression, trauma
30	Coping with Disasters and Pandemics Through Experience and Community: How African American Older Adults Navigate Disaster Planning, Response, and Recovery	Adepoju, O.E. and Smith, K.L. and Shetty, S. and Taha, E.-E. and Howard, D.L.	2023	Focus group interview	USA	N=26<br>Ethnicity: African American<br>Other particular identity: older adults who have spent most of their lives living in Houston	**Individual:** Regular health maintenance, chronic disease management; self-identified as active and social individuals; learned to use Zoom and Facebook Live to host meetings and gatherings; learning to live a new normal<br>**Micro:** Social connections; helpful information from community sources	being overwhelmed, fearful, frustrated
31	Intrusive rumination and academic burnout among adolescents in ethnic minority areas of China during the COVID-19 pandemic: PTSS as mediator and cognitive reappraisal as moderator	He, L. and Yuan, X. and Chen, Q. and Wang, X.	2023	Quantitative survey	China	N=791<br>Ethnicity: Tibetan and Qiang ethnic<br>Other particular identity: adolescents	**Individual:** Cognitive appraisal	Academic burnout

32	Contextualizing the experiences of Black pregnant women during the COVID-19 pandemic: ‘It’s been a lonely ride’	Dahl, A.A. and Yada, F.N. and Butts, S.J. and Tolley, A. and Hirsch, S. and Lalgondar, P. and Wilson, K.S. and Shade, L.	2023	Mix-method: online survey and Photo-Elicitation Interviews (PEI)	USA	N=40<br>Ethnicity: Black/ African American women<br>Other particular identity: prenatal women	**Individual:** Mediation; increased screen time (e.g., watching tv, video games, and smartphone use); eating more; doing physical activity; music listening; engaging in prayer or faith-based practices; decrease on drug use and alcohol<br>**Micro:** Talking with family, friends, and health providers; support from church community, online community	Stress
33	Commonalities and Differences in the Experiences of Visible Minority Transnational Carer–Employees: A Qualitative Study	Shahbaz, R. and Williams, A. and Sethi, B. and Wahoush, O.	2023	Qualitative interview	Canada	N=29<br>Ethnicity: Pakistani, Syrian, African, South American origin living in London<br>Other particular identity: Transnational Career–Employees	**Individual**: Keeping busy, praying, staying active<br>**Micro:** Family support	Overall mental well-being
34	Coping, Racial Discrimination, and Psychological Distress Among Asian American Parents	Fanta, A. and Kodama Muscente, K. and Kim, S.E. and Tsai, W. and Huang, C.Y.	2023	Online survey	USA	N=229<br>Ethnicity: Asian American	**Individual:** Indirect coping such as turning to work or other activities to take mind off things.	Psychological distress
35	Two sides of the same coin: A mixed methods study of Black mothers’ experiences with violence, stressors, parenting, and coping during the COVID-19 pandemic	Hassoun Ayoub, L. and Partridge, T. and Gómez, J.M.	2023	Mix-method: Online survey and qualitative interview	USA	N=127 in survey & N=118 in interview<br>Ethnicity: Black American	**Individual:** Turning to religion and prayer<br>**Micro:** Having more time with family or kids	Depression and anxiety
36	Structural Inequities in Self-compassion and Parental Burnout	Kroshus, E. and Hawrilenko, M. and Tandon, P.S. and Browning, A. and Steiner, M.K. and Christakis, D.A.	2023	Online or phone survey	USA	N=2324 (including ethnic White)<br>Ethnicity: Black, Asian, Hispanic, mixed ethnicity<br>Other particular identity: parents with at least one child aged 4-17	**Individual:** More self-compassion	Mental health difficulties such as emotional problems, peer problems, hyperactivity.

37	African American Women Breast Cancer Survivors: Coping with the COVID-19 Pandemic	Hamilton, J.B. and Abiri, A.N. and Nicolas, C.A. and Gyan, K. and Chandler, R.D. and Worthy, V.C. and Grant, E.J.	2023	Online or phone interview	USA	N=18<br>Ethnicity: African American<br>Other particular identity: African American Women breast cancer survivors	**Individual:** Seeking control in their social environments; following the treatment rules<br>**Micro:** Seeking support from God, family, and friends.	Stress
38	Revisiting the Black-White Mental Health Paradox During the Coronavirus Pandemic	LaMotte, M.E. and Elliott, M. and Mouzon, D.M.	2023	Quantitative survey	USA	N=594 (including ethnic White)<br>Ethnicity: African American	**Individual:** Higher self-esteem	Mental health outcome including psychological distress, somatic symptom burden, and prior diagnoses of depression, and anxiety.
39	"We lost a lot, but something good came out of it too:" Exploring the impact of the COVID- 19 pandemic on the mental wellbeing of British Muslim Pakistani women with family responsibilities	Iqbal, H. and Lockyer, B. and Iqbal, S. and Dickerson, J.	2023	Qualitative interview	UK	N=25<br>Ethnicity: Pakistani<br>Other particular identity: Hritish Muslim Pakistani women with family responsibilities	**Individual:** Islamic religious faith - coping through prayer and supplication; larger household structure; closeness to faith; achieve work-life balance<br>**Micro:** Closeness to family; generosity among neighbours<br>**Macro:** Islamic religious faith - belief in predestination	Overall mental health
40	Black adolescents' perceptions of COVID-19: Challenges, coping, and connection to family, religious, and school support.	Parker JS and Haskins N and Lee A and Hailemeskel R and Adepoju OA	2021	Qualitative interview	USA	N=18<br>Ethnicity: Black<br>Other particular identity: Black youth between the ages of 12 and 18 years	**Individual:** Emotion and problem-focused coping, religious/ spiritual in nature.<br>**Micro:** Social support from family, school personnel, and religious community (varied support received)	Overall mental health
41	Stress and coping among pregnant black women during the COVID-19 pandemic.	Wheeler JM and Misra DP and Giurgescu C	2021	Quantitative survey	USA	N=33<br>Ethnicity: Black<br>Other particular identity: Pregnant women	**Individual:** Obtain social media information, and following government advice<br>**Macro:** God, religion, or spirituality	Stress

42	Coping While Black: Comparing Coping Strategies Across COVID-19 and the Killing of Black People.	Cox JM and Toussaint A and Woerner J and Smith A and Haeny AM	2023	Quantitative analysis through online survey	USA	N=128<br>Ethnicity: Black	**Individual:** Active coping and self-distraction, prayer and meditation; trying to reframe changes in a positive light<br>**Micro:** Seeking emotional support from family and friends	Stress
43	The mental health experiences of ethnic minorities in the UK during the Coronavirus pandemic: A qualitative exploration	Van Bortel, T. and Lombardo, C. and Guo, L. and Solomon, S. and Martin, S. and Hughes, K. and Weeks, L. and Crepaz-Keay, D. and McDaid, S. and Chantler, O. and Thorpe, L. and Morton, A. and Davidson, G. and John, A. and Kousoulis, A.A.	2022	Qualitative interview	UK	N=30<br>Ethnicity: British Asian, British Black/African/Caribbean, multiple ethnic groups	**Individual:** Engaging in exercise<br>**Micro:** Keep in touch with friends and family; having support systems from different networks of life—friends, family, work, neighbors, religious community; attend religious services online<br>**Macro:** Have faith	lonely and isolated, anxiety and uncertainty,
44	Psychological Outcomes and Culturally Relevant Moderators Associated With Events of Discrimination Among Asian American Adults	Liu, M.A. and Prestigiacomo, C.J. and Karim, M.F.A. and Ashburn-Nardo, L. and Cyders, M.A.	2022	Online survey	USA	N=300<br>Ethnicity: Asian American	**Individual:** Self-esteem	Depressive symptom
45	COVID-19 Racial Discrimination and Mental Health of Korean Americans: Role of Ethnic Identity and Coping Strategy	Oh, S. and Litam, S.D.A. and Chang, C.Y.	2022	Quantitative survey	USA	N=270<br>Ethnicity: Korean American	**Individual:**<br>Coping strategies such as engagement coping, problem-focused engagement coping, problem-focused disengagement coping can moderate the relationship between depression and life satisfaction; ethnic identity also play a moderation role between racial discrimination and levels of anxiety and depression.	Anxiety and depression

46	The Impact of COVID-19 on Black College Students' Mental Health	Jones, M.K. and Leath, S. and Latimer, K. and Lawson, E. and Briones, M.	2022	Qualitative interview	USA	N=46<br>Ethnicity: Black<br>Other particular identity: Black college student	**Individual:** Spiritual; engagement coping, such as developing new hobbies<br>**Micro:** Seeking social support by finding innovative ways to communicate with friends or relied on their peers for venting and emotional support	Depressive symptom, stress, and anxiety
47	Cross-Sectional Study of Factors Influencing Perceived Threat and Stress among the Arab Minority in Israel during the COVID-19 Pandemic.	Ali-Saleh O and Halperin O	2022	Quantitative survey	Israel	N=486<br>Ethnicity: Arab	**Individual:**<br>Higher locus of control and problem-focused coping are the protection of stress.	Overall, a moderate-low level of stress and a moderate-high level of perceived threat were calculated.
48	Evaluating Experiences of Stress and Coping Among African American Women During the COVID-19 Pandemic to Inform Future Interventions.	Sweeney AM and Wilson DK and Zarrett N and Brown A and Quattlebaum M and Gorman B and Loncar H	2021	Qualitative interview	USA	N=17<br>Ethnicity: African American<br>Other particular identity: Women	**Individual:** Go outside; get in a regular routine; problem-focused coping approaches, including health behaviors, enjoyable activities (e.g., reading, listening to music, and arts/crafts), and identifying areas for improvement;<br>**Micro:** Emotional social support from family members such as sister; receiving support on child care; problem-focused coping strategies included quality time with family/friends and limiting TV/media; the role of church, virtual bible study and church services, church emails/newsletters, and staying connected with church members through calls/texts.	Stress in relation to COVID-19

49	A Phenomenological Study on the Positive and Negative Experiences of Chinese International University Students From Hong Kong Studying in the U.K. and U.S. in the Early Stage of the COVID-19 Pandemic.	Lai AY and Sit SM and Lam SK and Choi AC and Yiu DY and Lai TT and Ip MS and Lam TH	2021	Focus group interview	USA	N=20<br>Ethnicity: Chinese<br>Other particular identity: international students	**Individual:** Positive thinking and using alternative measures in meeting challenges<br>**Micro:** The importance of support from family, friends, and schools	Worries and stress; positive personal growth
50	Anti-Asian Microaggressions in the Time of COVID-19: Impact on Coping, Stress, and Well-Being	Yan, X. and Zhu, Y. and Hussain, S.A. and Bresnahan, M.	2022	Mix-method: Online survey and qualitative interview	USA	N=345 in survey & N=196 in interview<br>Ethnicity: East Asian	**Individual:** Strong personal resilience and engagement coping;<br>**Micro:** More social support	Psychological well-being
51	Ethnic Identity and Coping Strategies as Moderators of COVID-19 Racial Discrimination Experiences Among Chinese Americans	Litam, S.D.A. and Oh, S.	2022	Quantitative survey	USA	N=187<br>Ethnicity： Chinese	**Individual:** Strong ethnic identity; higher level of coping	Depression
52	Asian American Mental Health Amidst COVID-19 Anti-Asian Racism: Internalized Racism and Generational Status as Moderators	Liu, T. and Liu, C. and Chang, Y.J.	2022	Online survey	USA	N=565<br>Ethnicity: Asian American	**Individual**: Higher level of internalized racism among1.5 and 2nd+ generation Asian Americans.	Depression and anxiety symptom
53	Exploring Black adolescents' perceptions of God during COVID-19: God images as a source of wellness	Parker, J.S. and Williams, B. and Mauney, A.	2022	Interview	USA	N=11<br>Ethnicity: Black<br>Other particular ethnicity: adolescents age 12-17	**Macro:** God image as a source of capacity to thrive when faced with related challenges.	Stress

54	COVID-19 Racial Discrimination on Mental Health and Life Satisfaction Among Asian Americans: Examining a Moderated Mediation Model	Oh, S. and Litam, S.D.A.	2022	Quantitative survey	USA	N=725<br>Ethnicity: Asian Americans and Pacific Islanders	**Individual:**<br>High level of ethnic identity is also a protective factor which attenuated the relationship between depression and life satisfaction; engagement coping strategies and disengagement coping strategies show its positive effects on reducing depression and anxiety.	Depression, anxiety, and life satisfaction
55	Effects of COVID-19 on Adolescent Mental Health and Internet Use by Ethnicity and Gender: A Mixed-Method Study	Kaya, M.S. and McCabe, C.	2022	Mix-method: Online survey and qualitative interview	UK	N=108 in survey & N=32 in interview<br>Ethnicity: BAME	**Individual:** Use of social media to find new hobbies<br>**Micro:** Use of social media to make new friends; the supportive role of family	Depression and anxiety
56	Coping Matters: An Examination of Coping Among Black Americans During COVID-19	Mercier, C.M. and Abbott, D.M. and Ternes, M.S.	2022	Mix-method: Online survey and online interview	USA	N=155 for survey & interview<br>Ethnicity: Black American	**Individual:** Engagement in heathier behaviors; cooking and eating heathy foods; listening to music and reading; personal religious and practice<br>**Micro**: Increase social connection through phone calls and virtually through videoconferencing; engaging in other activities	Depressed, grief, loneliness, fear, anxiety,
57	COVID-19 conversations: A qualitative study of majority Hispanic/Latinx youth experiences during early stages of the pandemic	Cortés-García, L. and Hernández Ortiz, J. and Asim, N. and Sales, M. and Villareal, R. and Penner, F. and Sharp, C.	2022	Qualitative group interview	USA	N=17<br>Ethnicity: Hispanic/Latinx<br>Other particular identity: youth	**Individual:** Personal recognition; breathing to relax and outdoor activities<br>**Micro:** They rely on the members of the community and bond together; providing more help to others than before; close and open communication with family members; the use of video chatting (e.g., FaceTime) and social media (e.g., TikTok)	Two side emotions: loneliness, sadness and boredom; feelings of happiness and joy of being at home with their families and being less stressed.

58	Mental health experiences and coping strategies of BAME care workers who worked in nursing and residential care homes during the COVID-19 pandemic in Luton, England.	Kabasinguzi I and Ali N and Ochepo P	2023	In-depth interview	UK	N=15<br>Ethnicity: BAME<br>Other particular identity: care workers who worked in nursing and residential care homes	**Individual:**<br>Religious practices; keeping themselves busy, doing activities they were passionate about; following government guidelines on the prevention of COVID-19<br>**Micro:**<br>Seeing the service users happy and some participants managed through support that was offered by the government<br>**Macro:**<br>Belief in God	The COVID-19 intensified low mood, which can be encountered as depression, anxiety, and disordered eating.
59	Asian Americans' Racial Discrimination Experiences During COVID-19: Social Support and Locus of Control as Moderators	Lu, Y. and Wang, C.	2021	Quantitative survey	USA	N=218<br>Ethnicity: Asian/Asian American	**Individual:** Low external locus of control<br>**Micro:** Social support	Generalized anxiety and depressive symptom
60	Predicting psychological distress amid the COVID-19 pandemic by machine learning: Discrimination and coping mechanisms of Korean immigrants in the U.S.	Choi, S. and Hong, J.Y. and Kim, Y.J. and Park, H.	2020	Mix-method: Quantitative survey and machine learning	USA	N=790<br>Ethnicity: Korean	**Individual:** high resilience capacity	Psychological distress
61	Impacts of COVID-19 on African Migrants' Wellbeing, and Their Coping Strategies in Urban and Regional New South Wales, Australia: a Qualitative Study	James, PB and Gatwiri, K and Mwanri, L and Wardle, J	2023	Individual semi-structured in-depth interviews	Australia	N=21<br>Ethnicity: African	**Individual:** Holding on to religion and faith, indulging in self-care practices such as healthy eating, exercise, Yoga, meditation, sleep, and limited interaction with social media.<br>**Micro:** Community support from religious organizations	Stress, frustration, anxiety, sadness, loneliness, and depression.

62	"You have to continue doing the work": Black women essential workers coping amidst the dual pandemics of COVID-19 and racism	Godoy, SM and Dukes, L and Chapman, M and Day, S and Goode, RW	2023	Individual interviews	USA	N=22<br>Ethnicity: Black<br>Other particular identity: Black women as essential workers	**Individual:**<br>Overeating; alcohol consumption<br>**Micro:**<br>Avoiding difficult race-based discussions; Prioritization of work; Talking with friends; Getting social support through videoconferencing system;<br>**Macro:**<br>Faith and spirituality	Passive distress
63	Coping, COVID knowledge, communication, and HBCU student's emotional well-being: Mediating role of perceived control and social connectedness	Huang, Hsuan Yuan and Li, Huijun and Hsu, Ying-Chia	2022	Online survey	USA	N=254<br>Ethnicity: African American<br>Other particular identity: Students from historically Black College and University	**Individual:** Self-care coping strategies, feelings of being in control in life, mind−body balance coping strategies, be knowledgeable about COVID-19<br>**Micro:** Social connectedness, have constant communication with others	Emotional well-being
64	Racism and stress-related growth among Asian internationals: Ethnic identity, resilience, and coping during Covid-19	Oh, Seungbin and Litam, Stacey Diane Arañez and Chang, Catharina Y.	2022	Quantitative survey	USA	N=237<br>Ethnicity: Asian<br>Other particular identity: international students and workers	**Individual:**<br>Ethnic identity, coping (self-distraction, active coping, denial, positive reframing), and resilience (personal competence and acceptance of self and life)	Stress-related growth
65	Resilience and coping as moderators of stress-related growth in Asians and AAPIs during COVID-19	Litam, Stacey Diane Arañez and Oh, Seungbin and Chang, Catherine	2021	Quantitative survey	USA	N=326<br>Ethnicity: Asian Americans and Pacific Islanders	**Individual:**<br>Higher levels of coping, resilience, and ethnic identity	Stress-related growth
